# Examining prestructured β-actin peptides as substrates of histidine methyltransferase SETD3

**DOI:** 10.1038/s41598-024-76562-z

**Published:** 2024-11-02

**Authors:** Marijn N. Maas, Nurgül Bilgin, Laust Moesgaard, Jordi C. J. Hintzen, Anna Drozak, Jakub Drozak, Jacob Kongsted, Jasmin Mecinović

**Affiliations:** 1 Department of Physics, Chemistry and Pharmacy, University of Southern Denmark, Campusvej 55, 5230 Odense, Denmark; 2 Department of Molecular Plant Physiology, Faculty of Biology, University of Warsaw, Miecznikowa 1, 02-096 Warsaw, Poland; 3 Department of Metabolic Regulation, Faculty of Biology, University of Warsaw, Miecznikowa 1, 02-096 Warsaw, Poland

**Keywords:** Chemical modification, Chemical tools, Enzymes, Peptides, Chemical biology, Chemistry

## Abstract

The N^τ^-His73 methylation of β-actin by histidine methyltransferase SETD3 is required for the integrity of the cellular cytoskeleton. Modulation of SETD3 activity in human cells facilitates cancer-like changes to the cell phenotype. SETD3 binds β-actin in an extended conformation, with a conserved bend-like motif surrounding His73. Here, we report on the catalytic specificity of SETD3 towards *i*,* i + 3* stapled β-actin peptides possessing a limited conformational freedom surrounding the His73 substrate residue via positions Glu72 and Ile75. Stapled β-actin peptides were observed to be methylated less efficiently than the linear β-actin peptide. None of the stapled β-actin peptides efficiently inhibited the SETD3-catalyzed N^τ^-His73 methylation reaction. Molecular dynamics simulations demonstrated that the unbound and SETD3-bound β-actin peptides display different backbone flexibility and bend-like conformations, highlighting their important role in substrate binding and catalysis. Overall, these findings suggest that reduced backbone flexibility of β-actin prevents the formation of optimal protein-peptide interactions between the enzyme and substrate, highlighting that the backbone flexibility needs to be considered when designing β-actin-based probes and inhibitors of biomedically important SETD3.

**Supplementary Information:**

The online version contains supplementary material available at 10.1038/s41598-024-76562-z.

## Introduction

The cytoskeleton of the cell consists of actin protein microfilaments, which play a crucial role in maintaining cytoskeletal stability, while also facilitating processes such as cellular motility, phagocytosis, cell contraction, organelle transport and signaling^1,2^. The human genome encodes six highly conserved and abundant actin isoforms with tissue specific expression patterns; α*-*skeletal, α*-*cardiac, α*-*smooth, β-cytoplasmic, γ-cytoplasmic, and γ-smooth^3–5^. Globular β-actin (βA) monomers are expressed ubiquitously in human cells and polymerize upon binding of ATP to form filaments (F-actin), which maintain cellular stability and facilitate cellular mobility^1,6,7^. Actin filament depolymerization is mediated by hydrolysis of bound ATP, resulting in a dynamic interplay controlling the length of actin filaments in the cell^8,9^.

**Fig. 1:**
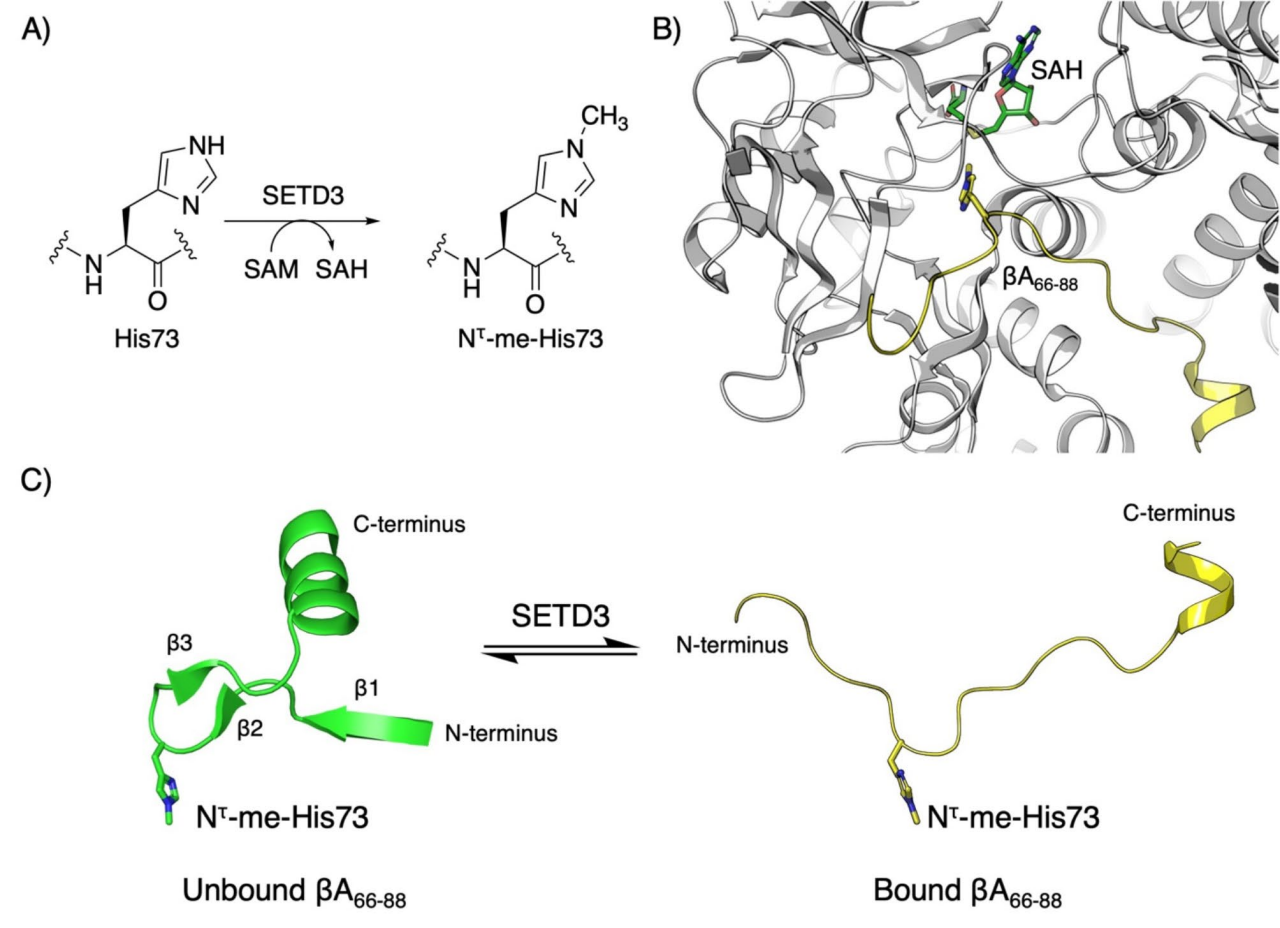
β-Actin as a substrate for histidine methyltransferase SETD3. (**A**) SETD3-catalyzed N^τ^-methylation of His73 in βA. (**B**) View on the crystal structure of SETD3 (grey) complexed with methylated β-actin (yellow) and SAH (green) (PDB: 6ICT). (**C**) A representation of structural change in βA upon SETD3 binding. Comparison of βA in native conformation in the unbound form (green) and a SETD3-bound conformation (yellow) (PDB: 1HLU, 6ICT).

Actin proteins are subject to various post-translational modifications (PTMs), such as methylation, acetylation, ubiquitination and SUMOylation, which affect the cytoskeletal dynamics^9,10^. Members of the Su(var)3–9, Enhancer of zeste, and Trithorax (SET) domain family commonly deposit methyl moieties from the *S*-adenosylmethionine (SAM) co-substrate on lysine or arginine residues via an S_N_2 reaction, generating *S*-adenosyl-L-homocysteine (SAH) as a co-product^11,12^. Like other methyltransferases belonging to the SET-domain family, SETD3 was originally proposed to catalyze methylation of histone H3 at K4 and K36 sites^13,14^. However, SETD3 was recently shown to specifically catalyze N^τ^-methylation of His73 in βA (βA-H73) in human and Drosophila cells (Fig. 1A)^3,15,16^. This conserved PTM is associated with a decreased rate of βA-bound ATP hydrolysis, resulting in more stable actin filaments^3^. Loss of N^τ^-methylation of βA in human HAP1 cells was shown to induce cancer-like changes to the cell phenotype due to actin protein microfilament depolymerization, leading to loss of cytoskeletal integrity and increased glycolysis-mediated ATP consumption. This finding suggests a role of SETD3 in tumor suppression^16^.

Structural analysis of SETD3 revealed that, similar to other SET methyltransferases, SETD3 consists of an N-SET, catalytic SET, insertion SET and a C-terminal substrate-binding domain^3,17,18^. Crystal structures of SETD3 in complex with SAH and a βA substrate fragment peptide reveal that target specificity for βA is mediated by an electrostatically bipartite groove on the protein surface formed by the SET, insertion SET and C-terminal domains (Fig. 1B)^3,17,18^. The nucleophilic N^τ^ atom of His73 in βA is angled towards the methyl group of SAM in a linear arrangement, to mediate the transition state required for the SETD3-catalyzed S_N_2 reaction. Additional sequence selectivity is obtained through secondary hydrophobic βA binding sites of SETD3 that accommodate the binding of βA-I71 and βA-W79 residues^15,19,20^. The local secondary structure of residues 66–84 of unbound βA comprises three β strands (β1-β3) of which β2-β3 introduce a local bend-like conformation, followed by an α-helix. Binding of βA by SETD3 requires native βA to decompose its local secondary structure to adopt an extended conformation (Fig. 1C), which facilitates an extensive network of intra- and intermolecular hydrogen bonding and hydrophobic interactions with the SETD3 surface^3^. However, a hydrogen bonding interaction between the backbone carbonyl of βA-E72 and the backbone amide of βA-I75 is observed to conserve the bend-like conformation of the βA backbone (Fig. 1C), which allows for optimal positioning of βA-H73 into the hydrophobic active-site channel of SETD3. SETD3 apparently requires an assistance of factors such as peptide release factor eRF3a to unfold βA into a methylation-compatible conformation^3,16^. The apparent importance of the local bend-like conformation surrounding His73 in βA has prompted us to investigate here the importance of βA backbone flexibility required for efficient recognition and catalysis by human SETD3 methyltransferase through chemically constrained βA peptide substrates. We have recently demonstrated that structurally and chemically diverse histidine mimics are accepted as SETD3 substrates^21^. In addition, we have shown that substitution of βA-H73 with methionine analogs such as selenomethionine in βA peptide fragments can result in highly potent SETD3 inhibitors^22^. βA-I71 and βA-W79 substitutions with analogs of isoleucine and tryptophan, respectively, revealed that ‘secondary’ Ile71 and Trp79 binding sites play a significant role in modulating βA methylation efficiency^23,24^. Additional insight into the importance of the βA-backbone flexibility could provide important functional and mechanistic knowledge into the methylation of βA-H73 by SETD3.

## Results and discussion

**Fig. 2:**
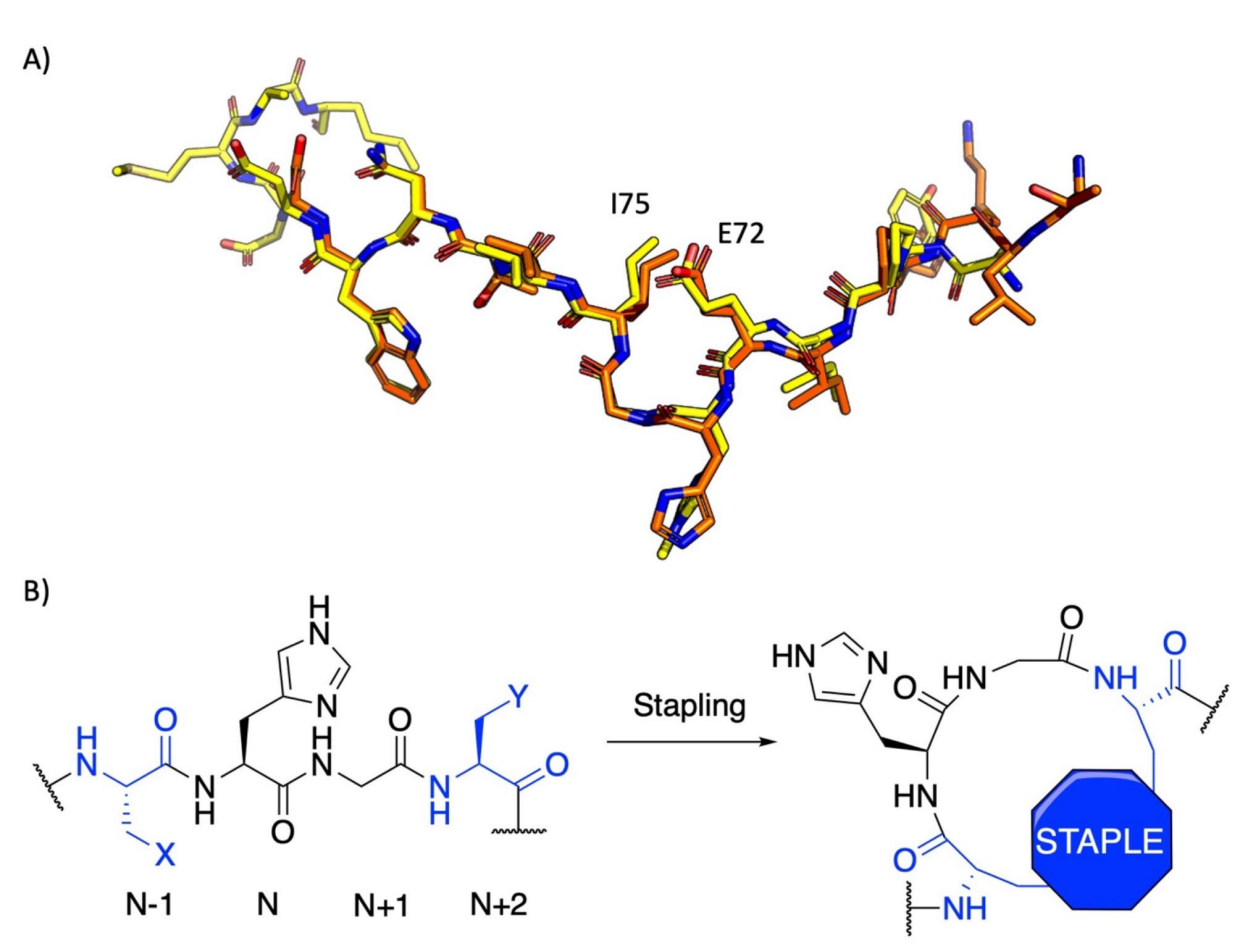
Stapling of βA peptides. (**A**) Overlay of βA structures in liquorice representation bound to SETD3. βA-H73 (orange, PDB-ID: 6MBK) and βA-N^τ^-me-H73 (yellow, PDB-ID: 6ICT) share a similar overall binding conformation. (**B**) Stapling *i*,* i + 3* strategy to constrain the secondary structure of βA in a β-turn-like conformation. X and Y represent residue side chains, which can be selectively stapled during peptide synthesis.

We hypothesized that limiting the available conformations of βA through stapling might enhance binding affinity and selectivity for SETD3-catalyzed methylation of βA-H73. Structural data of SETD3 in complex with βA suggest that stapling through direct covalent linking might facilitate stabilization of the bioactive bend-like conformation found in βA, while keeping key recognition elements of the βA peptide sequence undisturbed^25,26^. We selected peptide *i*,* i + 3* stapling sites Glu72 and Ile75 to replace the transient side chain noncovalent interactions with a covalent linkage through stapling. In the SETD3-βA-SAH complex, the selected residues appear to be in close proximity to each other while in the bend-like conformation (distance between side chain terminals: 4.57–5.04 Å). Glu72 and Ile75 side chains are directed away from the SETD3 surface, suggesting that substitution might not interfere with the binding interface (Fig. 2A)^3,18,23^. In addition, *i*,* i + 3* stapling has been used to stabilize non-regular secondary structures, like β-turns, in peptides with key recognition hotspots^25^. A panel of conformationally restrained βA_66-81_ peptides was synthesized employing three common strategies for side chain-to-side chain stapling to introduce varying levels of conformational restriction, steric-bulk, lipophilicity or electrostatic character to the cyclic βA peptides (Fig. 2B); bis-thiol alkylation^27^, lactam-bridge formation^28,29^, and azide-alkyne Cu^I^-mediated Huisgen 1,3-dipolar cycloaddition^30^.

**Fig. 3:**
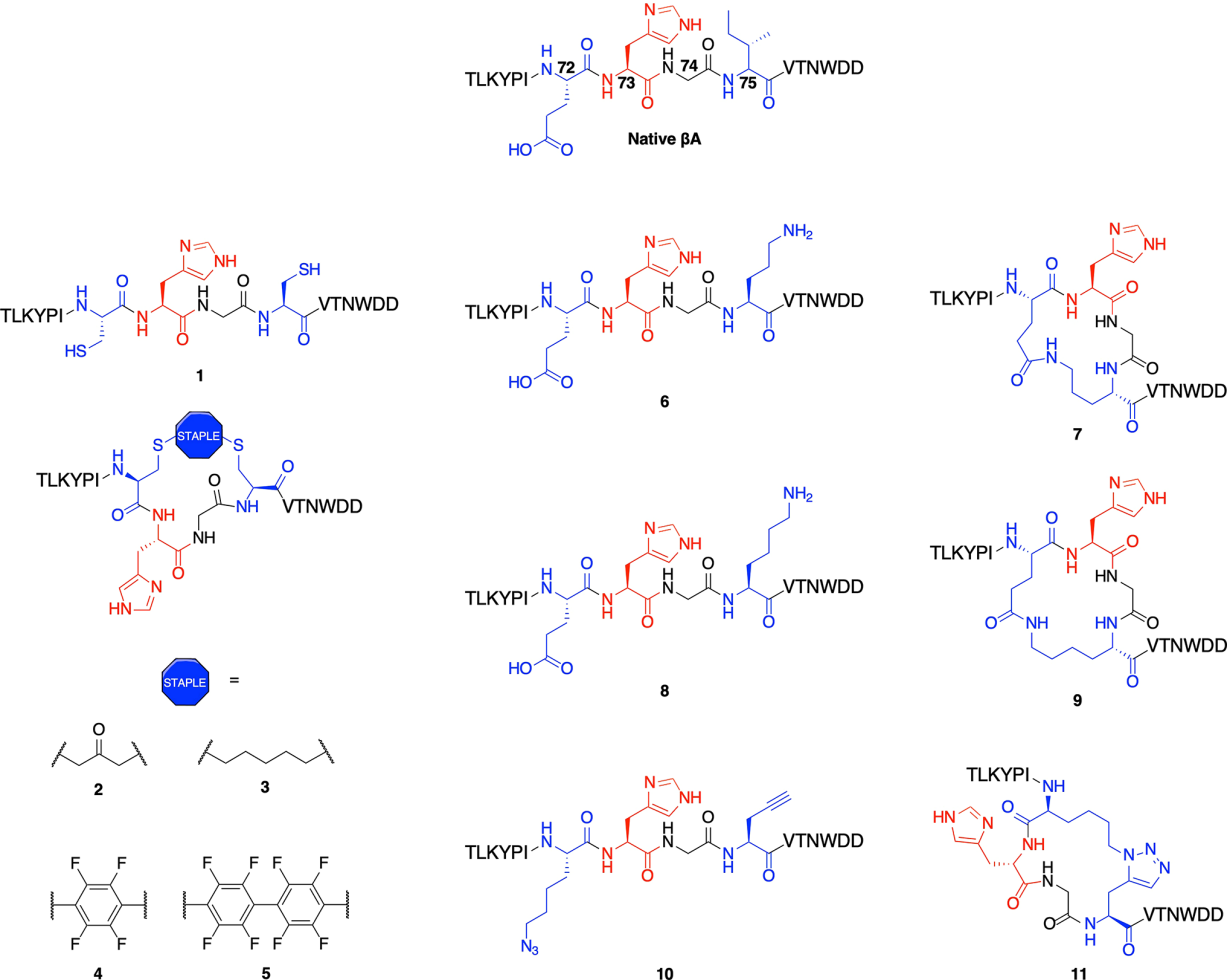
Design of stapled βA_66-81_ peptides as substrates of SETD3. Linear modified peptides **(1**,** 6**,** 8**,** 10)** were used as controls to their stapled counterparts **(2**,** 3**,** 4**,** 5**,** 7**,** 9**,** 11**). One-letter codes for L-amino acids are used.

A panel of conformationally restrained βA_66-81_ peptides and their linear controls **1**–**11** was generated with standard Fmoc-based solid-phase peptide synthesis (SPPS) and subsequent side chain-to-side chain covalent crosslinking following established procedures (Fig. 3)^27–30^. Linear controls were synthesized to evaluate the effect of peptide stapling on recognition and enzyme catalysis, as the functional groups introduced by stapling might engage in separate protein-peptide interactions. Mass spectrometry and analytical HPLC confirmed the high purity (> 90%) of all RP-HPLC purified βA peptides (Table S1 and Figs. S1–S2).

**Fig. 4:**
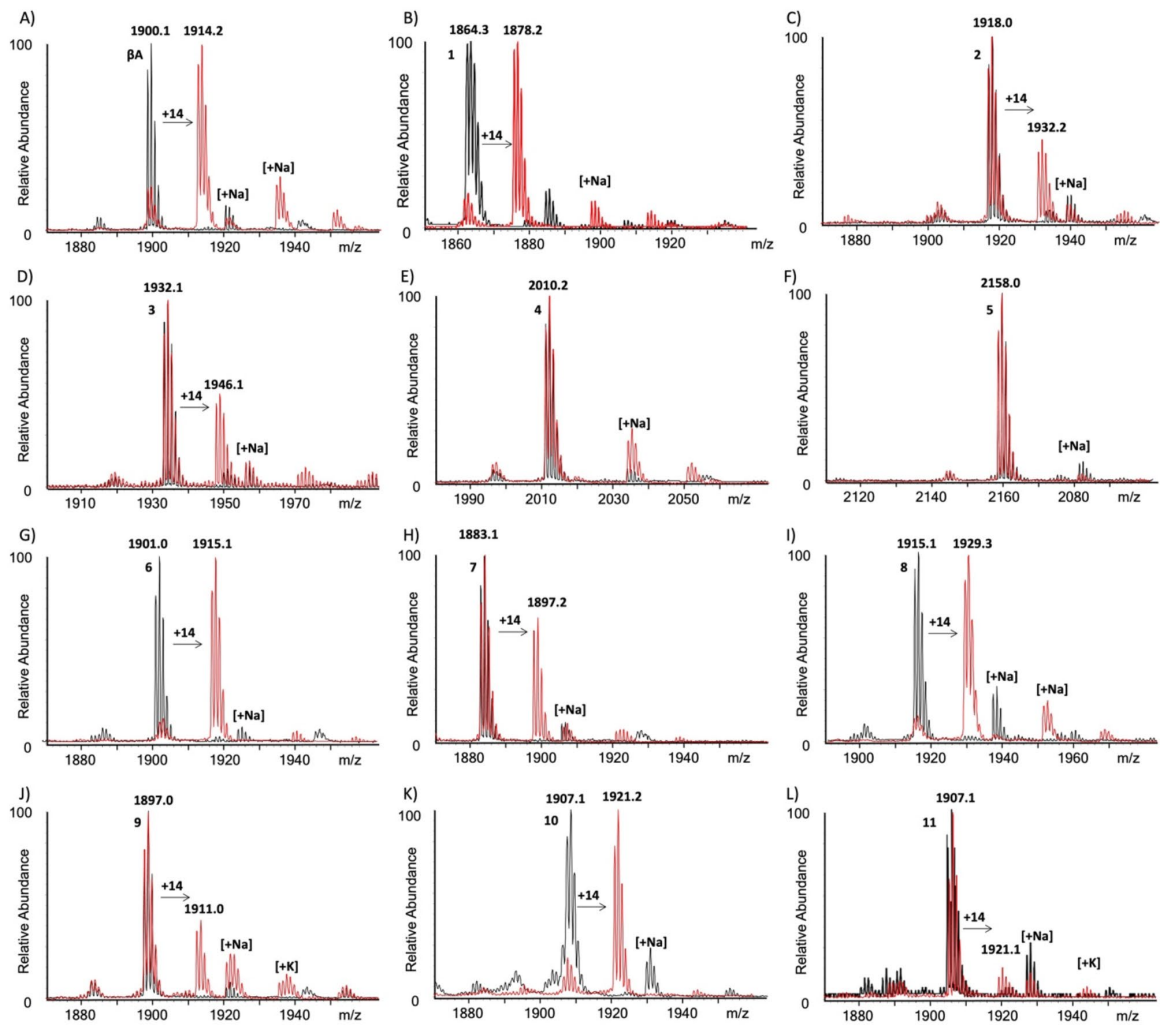
MALDI-TOF MS data showing SETD3-catalyzed (1 µM) methylation of βA peptides (10 µM) in the presence of SAM (100 µM) in reaction buffer (25 mM Tris, 20 mM NaCl, pH = 9.0) after 3 h at 37 °C. (**A**) βA, (**B**) **1**, (**C**) **2**, (**D**) **3**, (**E**) **4**, (**F**) **5**, (**G**) **6**, (**H**) **7**, (**I**) **8**, (**J**) **9**, (**K**) **10**, (**L**) **11**. Control reactions in the absence of SETD3 are shown in black, whereas SETD3-catalyzed reactions are shown in red.

We examined conformationally restrained βA peptides and linear controls as substrates for the recombinantly expressed human SETD3 employing MALDI-TOF MS to detect a characteristic + 14 Da mass increase upon methylation. Enzymatic reactions were evaluated using standard conditions (1 µM SETD3, 10 µM βA, 100 µM SAM) in methylation buffer (25 mM Tris, 20 mM NaCl, pH = 9.0 at 37 °C)^21^. The enzymatic reaction was quenched with 10% TFA in MilliQ water (1:1 v/v) at 1 h and 3 h before mass spectrometric analysis (Figs . 4 and S3-S4). Control experiments using the natural βA-H73 sequence demonstrated full conversion within 1 h (Figs.4A and S4A). The enzymatic assays revealed that dual substitution of βA-E72 and βA-I75 by cysteine **(1)** is tolerated well by SETD3 under standard conditions, with full conversion being observed within an hour (Fig. S4B). Similarly, linear lactam controls **6** and **8**, which substitute βA-I75 with a primary amine (Orn/Lys) were completely methylated within an hour (Fig. S4G, I). Click-control **10**, however, reached full conversion only after 3 h incubation (Figs. 4K and S4K), indicating that βA possessing azido-lysine and propargylglycine is a poorer SETD3 substrate than the linear βA-H73 peptide. Stapled bis-thiol alkylated βA-peptides **2** and **3** were observed to be comparatively poorer substrates when compared to the wild-type sequence as well as the dicysteine variant (**1**), with 30% and 35% of methylation being observed after 3 h, respectively (Figs. 4C, D and S4C, D). Stapling with hexafluorobenzene **(4)** or decafluorobiphenyl **(5)** rendered the cyclic βA-peptides inert to methylation by SETD3 (Figs. 4E, F and S4E, F), suggesting that the flexibility present in the macrocycle of **2** and **3** is preferred over the rigid and bulky rings present in **4** and **5**. Introduction of an *i*,* i + 3* lactam-bridge to the βA-sequence yields peptide **7**, which was observed to be 41% methylated within 3 h (Figs. 4H and S4H). Increasing the macrocycle size by one atom **(9)** resulted in a poorer substrate (25% conversion after 3 h) compared to **7** or the native sequence, suggesting that the shorter ring size of **7** is better accommodated by SETD3 (Figs. 4J and S4J). Finally, azide-alkyne Cu^I^-mediated Huisgen 1,3-dipolar cycloaddition yields peptide **11** that was poorly (14%) methylated by SETD3 after 3 h under standard conditions (Figs. 4L and S4L). This result suggests that the rigidity and bulk introduced by the triazole is poorly tolerated by SETD3, since the macrocycle ring-size of **11** is equal (17 atoms) to that of the decent substrate **7**. Taken together, these findings illustrate an overall preference of human SETD3 for flexible βA peptides, which facilitates βA-H73 insertion into the active site. Introduction of flexible macrocycles results in an overall reduced methylation by SETD3, while bulky or rigidifying moieties present within the macrocycles render the βA peptides non-reactive to methylation by SETD3. The preference for SETD3 modifying the linear βA over stapled βA peptides was also verified by isothermal titration calorimetry (ITC) experiments, which showed that SETD3 binds the linear βA peptide with K_D_ = 2.46 µM, whereas the cyclic lactam peptide **9** displays significantly weaker binding affinity (K_D_ = 24.0 µM) (Fig. S5).

**Fig. 5:**
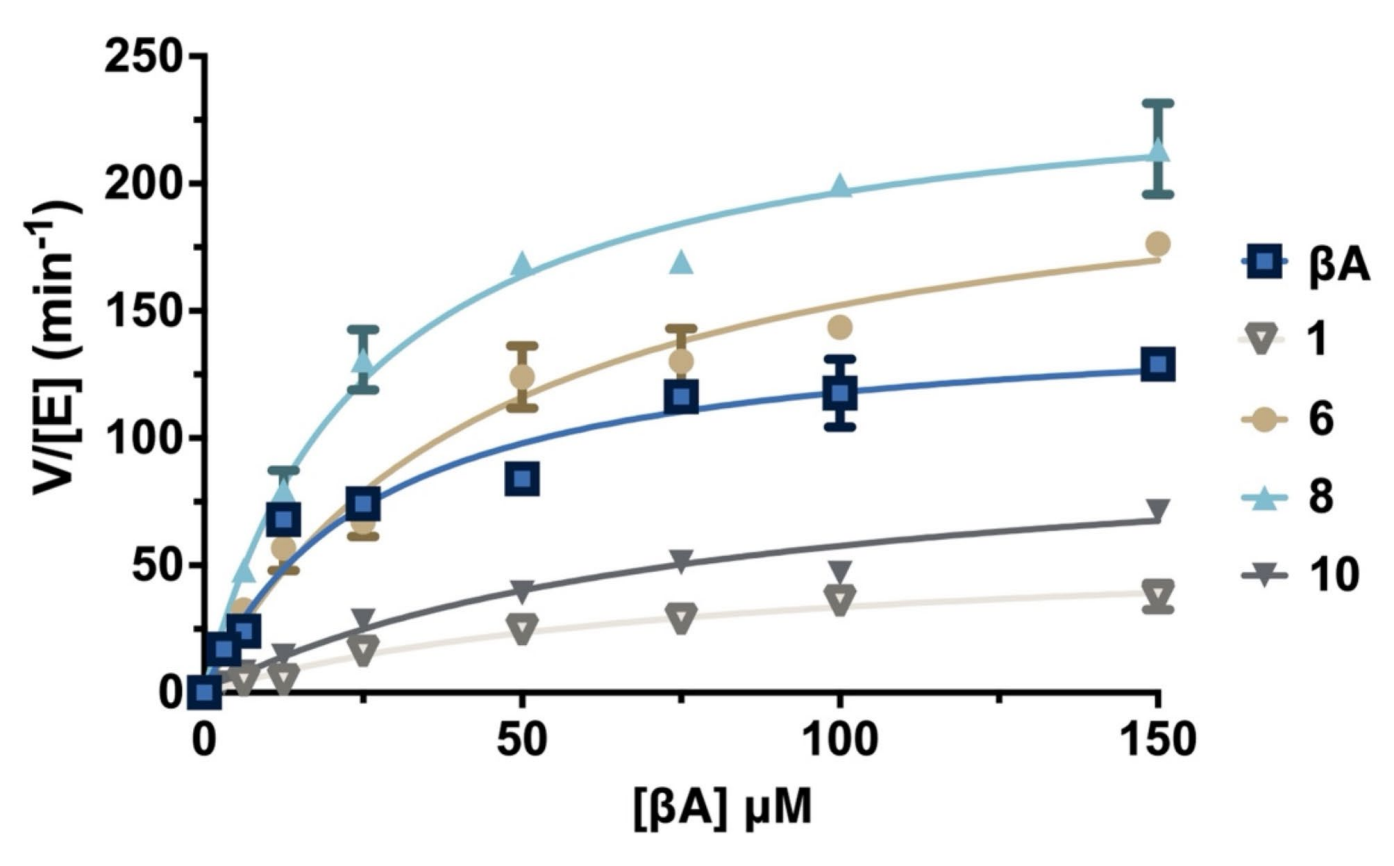
Michaelis–Menten kinetics of linear βA peptides at varying concentrations (3.125-150 µM) by SETD3 (200 nM) in presence of saturating amounts of SAM (100 µM) in reaction buffer (25 mM Tris, 20 mM NaCl, pH = 9.0) at 37 ^o^C.

Catalytic efficiencies were explored for SETD3-catalyzed methylation of linear βA peptides **1**,** 6**,** 8** and **10** (Fig. 5; Table 1). Kinetic parameters were determined using MALDI-TOF MS assays under steady-state conditions by incubation with saturating amounts of SAM (100 µM)^23^. Addition of SETD3 (200 nM) to varying concentrations of linear βA peptides (0–150 µM) in reaction buffer initiated the reaction, upon which the mixture was incubated at 37 °C and quenched at timepoints that guarantee v_0_-reaction rates for SETD3-catalyzed methylation. Direct comparison between relative catalytic efficiencies (k_cat_/K_m_) for SETD3-catalyzed methylation of βA peptides illustrates that substitution of βA-I75 by ornithine (Orn) **(6)** results in a comparable substrate to the native βA-H73 sequence. Interestingly, the change from aliphatic residue Ile75 to Lys75 **(8)** results in a 1.7-fold increase in catalytic efficiency, which is driven by a favorable increase in catalytic reaction rate (k_cat_) off-set by a less favorable K_m_. In contrast, double variants **1** and **10** were observed to be substantially worse substrates compared to the native sequence, both driven by a decrease in k_cat_ and an increase in K_m_ values. This result might suggest that substitution of βA-E72 is generally unfavorable due to the loss of a hydrogen bond acceptor, or that the introduction of propargylglycine and azidolysine, or cysteines result in unfavorable interactions with the surface of SETD3.

**Table 1:** Kinetics parameters for SETD3-catalyzed methylation of βApeptides.

Peptide	k_cat_ (min^-1^)	K_m_(µM)	k_cat_/K_m_ (µM^-1^min^-1^)
**βA**	148 ± 8.7	25.6 ± 4.9	5.78
**1**	59.0 ± 7.5	75.7 ± 20	0.78
**6**	221 ± 16	45.2 ± 8.1	4.91
**8**	246 ± 9.5	25.1 ± 3.1	9.80
**10**	103 ± 9.0	77.8 ± 14	1.32

**Fig. 6:**
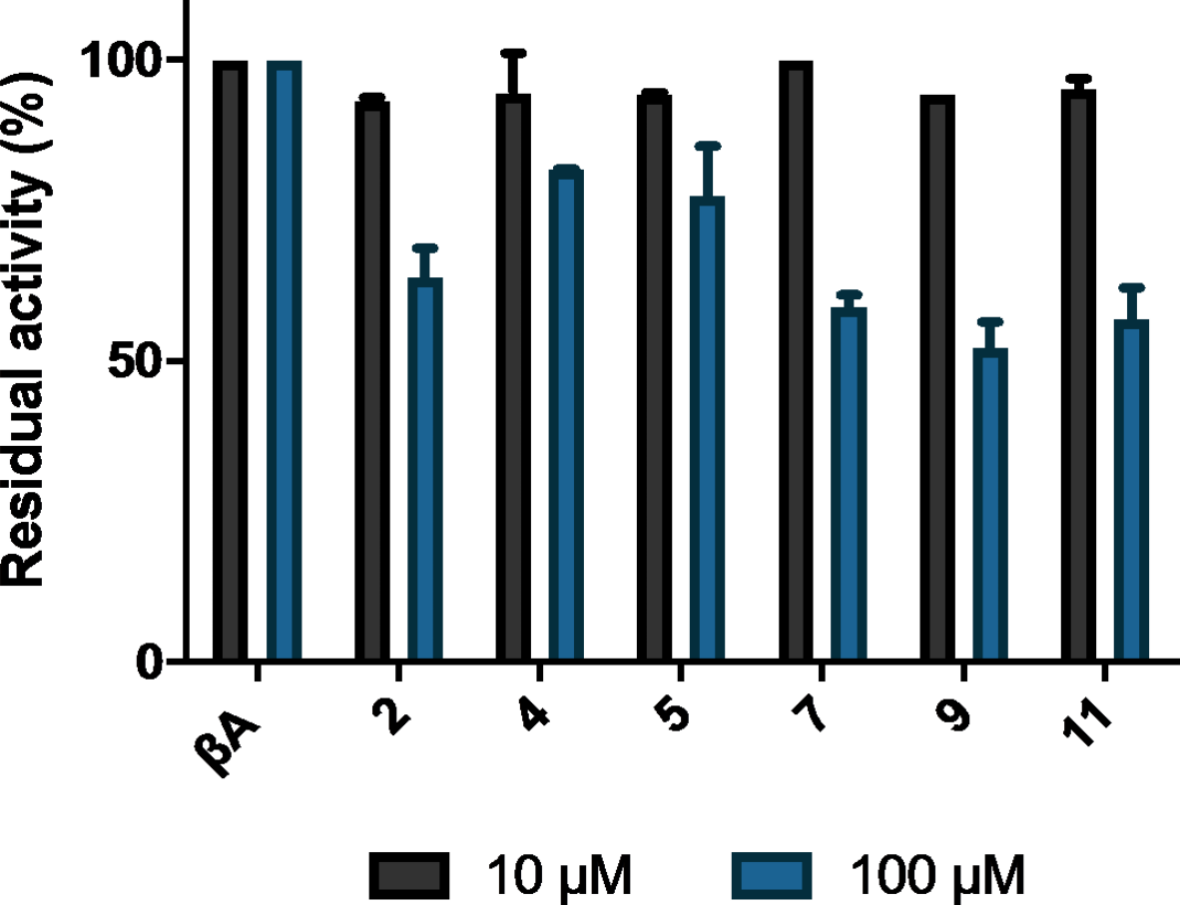
SETD3 inhibition assays. Single point inhibition assay of SETD3 (360 nM) by βA peptides (10 and 100 µM). Error bars reported as standard error (SE), *N* = 2.

Upon observing no or poor methylation of *i*,* i + 3* stapled βA peptides by SETD3, we carried out MALDI-TOF MS inhibition assays to evaluate the inhibitory potential of these peptides. For the initial single point assays, *i*,* i + 3* stapled βA peptides (10 and 100 µM) were preincubated with SETD3 (360 nM) and SAM (100 µM) in reaction buffer (25 mM Tris, 20 mM NaCl, pH = 9.0) at 37 ^o^C for 20 min, upon which the βA-H73 peptide was added to the mixture. The reaction was quenched after 20 min by addition of 10% TFA in MilliQ water (1:1, v/v) and aliquots were analyzed by MALDI-TOF MS (Fig. 6)^22^. Inhibition data revealed that none of the stapled βA peptides acts as an inhibitor of SETD3, with residual activities greater than 50% (IC_50_ > 100 µM) being quantified for all analogs when compared to the positive control reaction in the absence of a stapled βA peptide. The inhibitory potential for stapled βA peptides seems limited when compared to the recently reported βA-SeM73 inhibitor of SETD3 (IC_50_ = 161 nM)^22^.

To further study the dynamics of the βA peptide on an atomistic level, we performed multiple series of molecular dynamics (MD) simulations for βA-I75 (peptide βA), βA-**9** (peptide **9**), and βA-K75 (peptide **8**) unbound and bound to SETD3. For each of the six setups, eight replica of 100 ns were performed to thoroughly sample the motions of the peptides. From the simulations of βA-**9**, we observed that cyclization introduced the desired stability in the hairpin structure. Both the bound and unbound forms of βA-**9** maintained a stable Cα-Cα distance of approximately 6 Å between Glu72 and Lys75 (Fig. S6). Additionally, since the cyclization did not disrupt the positioning of other residues within the peptide, we hypothesize that the cyclization affects the binding process, rather than impairing the peptide’s ability to fit inside the SETD3 binding site, further supporting the thermodynamic binding analyses.

**Fig. 7:**
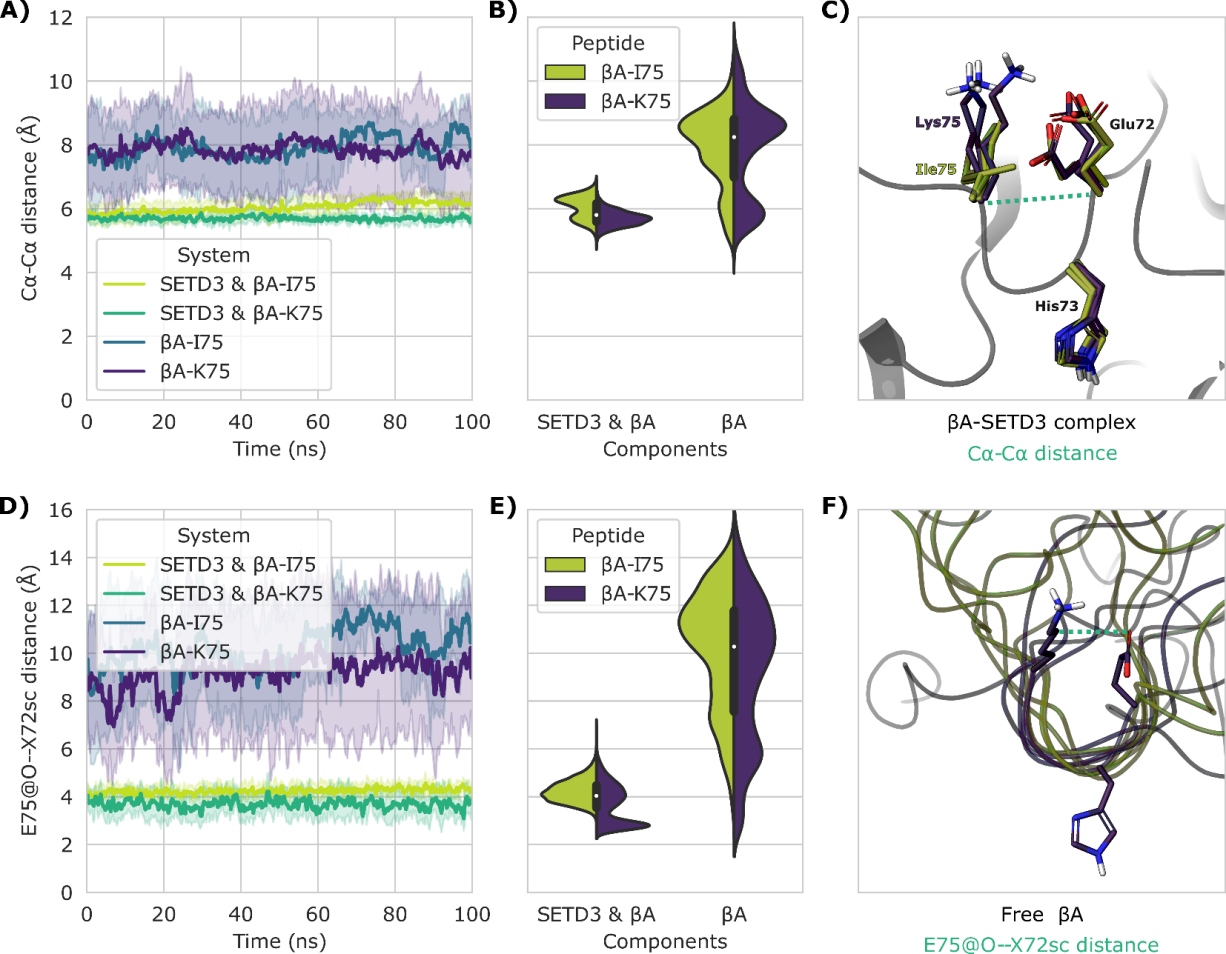
MD simulations of βA-I75 and βA-K75 in free and SETD3-bound forms. (**A**–**C**) The Cα-Cα distance between Glu72 and X75. D-F) The shortest distance from the carboxylic oxygens of Glu72 to any side chain (SC) atom of X75. (**A**, **D**) Plot of the mean distance over time. Shaded area is the standard deviation. (**B**, **E**) Violin-plot of the distribution of distances in (**A**, **D**). (**C**, **F**) Clustered conformations of the βA peptide during the MD simulations. Yellow: βA-I75, purple: βA-K75, green dashed line: distance reported in (**A**, **B**, **D**, **E**).

From the simulations of the non-lactam peptides, we also observed that the local bend-like conformation surrounding His73 remained relatively stable during the MD simulations of βA bound to SETD3 (Fig. 7A–C). However, a small average increment of 0.4 Å in the Cα-Cα distance between Glu72 and Ile75 was observed during the MD simulation of βA bound to SETD3, which resulted in a bimodal distribution of the distances. Interestingly, this was not observed in the simulation of the βA-K75 variant, which is likely due to the salt-bridge formed between Glu72 and Lys75. As the side chains of Glu72 and Ile75 remained in proximity throughout all simulations of βA bound to SETD3 (Fig. 7D–F), it is not surprising that this salt-bridge was observed to be relatively stable in all the simulations of SETD3 bound to the βA-K75 variant (Fig. S7). Additionally, Glu72 was observed to participate in a salt-bridge with Arg316 in all simulations with SETD3, which might explain why the peptides that retain the negative charge on Glu72 seemed to perform well in experimental assays (Fig. S7).

In the simulations of the unbound non-cyclized βA peptides, the peptides were observed to repeatedly form a hairpin conformation, thus decreasing the distance between the two terminals (Fig. S8). In this process, the hydrophobic residues on opposite sites are brought into proximity, which is likely the energetic driving force for the hairpin formation. In most cases, His73 is oriented towards the water phase, which likely benefits the recognition and binding to SETD3 (Fig. 7F). Interestingly, the bend-like conformation observed in the crystal structure is more frequently formed in the βA-K75 variant simulations (Fig. 7B), which could suggest that the I75K substitution primes the unbound peptide for SETD3 binding. This property could be contributing to the increased turnover of the βA-K75 peptide.

## Conclusion

With the recent identification of SETD3 as a methyltransferase of βA-H73, it becomes essential to investigate the underlying molecular principles that govern SETD3 recognition and catalysis. Better understanding of the flexibility of the βA backbone provides important functional and mechanistic insight into the methylation of βA-H73 by SETD3. Our enzymatic data illustrate that Glu72-Ile75 stapled βA peptides with restricted backbone flexibility are neither competent substrates nor inhibitors for SETD3-catalyzed methylation of βA-H73 when compared to their linear controls. Among the various stapled βA peptides, lactam **7** was methylated most efficiently, indicating that short flexible linkers are preferred over rigid and bulky analogs. This finding suggests that the βA backbone flexibility is required to accommodate optimal protein-protein interactions between the βA and SETD3’s substrate recognition domain. Taken together, the obtained results indicate that the flexibility of βA peptides should be retained when designing probes or inhibitors targeting SETD3. Continued exploration of biomolecular recognition and biocatalysis of βA substrates by SETD3 will provide an understanding of the role of specific protein-protein interactions in substrate recognition and methylation, aiding to the development of highly active and selective small molecule SETD3 inhibitors.

## Materials and methods

### General solid-phase peptide synthesis methods

βA peptides were chain assembled on Rink amide resin (100–200 mesh, loading = 0.40–0.80 mmol/g) using standardized SPPS reaction conditions. Amino acid couplings were carried out in presence of protected amino acid (3.0 eq.), HATU (3.0 eq.) and DIPEA (5.5 eq.) in DMF for 30 min on a rotating-mixer RM-5 (CAT Zipperer, Staufen, Germany) at RT. Full conversion is monitored using the Kaiser test method. Fmoc was removed by the addition of 20% piperidine in DMF for 30 min on a rotating-mixer at RT, of which full deprotection is monitored by Kaiser test. Upon completion of the linear peptide sequence, the peptide was treated with 20% piperidine in DMF for 30 min on a rotating-mixer at RT, of which deprotection was monitored by Kaiser test. The resin was subsequently washed extensively with DCM (3x), MeOH (3x) and Et_2_O to yield a dried resin before cleavage from resin for 4 h on a rotating-mixer at RT using a cleavage cocktail of TFA/TIS/H_2_O (95/2.5/2.5, v/v). Ice cold Et_2_O was subsequently added to the TFA solution to yield a milky white suspension, which was subjected to centrifugation at 4 °C in an Eppendorf 5804R centrifuge (Eppendorf, Hamburg, Germany) for 5 min at 5000 rpm. The supernatant was decanted and the remaining solid was resuspended in ice cold Et_2_O and centrifugated for 5 min at 5000 rpm. The solid crude peptide salt was dissolved in MilliQ H_2_O (10 mg/mL) for purification by preparative HPLC (Table S1 and Figs. S1–S2).

### Bis-thiol alkylation of βA peptides

Crude deprotected peptide **1** (1 mg, ~ 0.5 µmol, 1 eq.) was dissolved in 1 mL of cross-linking buffer (20 mM ammonium bicarbonate, pH = 8.0 in MilliQ H_2_O: ACN, 1:1, v/v). Linker (0.75 µmol, 1.5 eq.) in ACN (0.5 mL) was added to the peptide solution and the solution was shaken at 40 ^o^C at 750 rpm in an Eppendorf Thermomixer C. Reaction progress was monitored using MALDI-TOF MS and quenched with 10% TFA in MilliQ H_2_O (1:1 volumes) upon completion. A VaCo 2 lyophilizer (Zirbus Technology GmbH, Bad Grund, Germany) was used for lyophilization of the stapled peptides, upon which the solid crude peptide salt was dissolved in MilliQ water (10 mg/mL) for purification by preparative HPLC.

### Lactam-bridge formation of βA peptides

Fully protected linear βA peptides on rink amide resin with incorporated alloc- and allyl-protected Fmoc-lysine/Fmoc-ornithine and -glutamic acid, respectively, were swollen in presence of DCM under a flow of N_2_ for 10 min at RT. To the bubbling resin, phenyl silane (24 eq.) and tetrakis(triphenylphosphine)palladium (Pd(PPh_3_)_4_) (1 eq.) were added. The mixture was subjected to continued agitation under N_2_ flow for 30 min at RT to orthogonally remove alloc/allyl. Full conversion was monitored using a Kaiser test, before washing the resin with DCM (3x), DMF (3x) and sodium diethyldithiocarbamate (0.5% in DMF). Peptides requiring stapling were treated with HATU (3 eq.) and DIPEA (5.5 eq.) in DMF overnight on a rotating-mixer. Completion of stapling was monitored with a Kaiser test. Upon completion of the peptide sequence, the peptide was treated with 20% piperidine in DMF for 30 min on a rotating-mixer at RT, of which deprotection is monitored by Kaiser test. The resin was subsequently washed extensively with DCM (3x), MeOH (3x) and Et_2_O to yield a dried resin before cleavage from resin for 4 h on a rotating-mixer at RT using a cleavage cocktail of TFA/TIS/H_2_O (95/2.5/2.5). Ice cold Et_2_O was subsequently added to the TFA solution to yield a milky white suspension, which was subjected to centrifugation in in an Eppendorf 5804R centrifuge for 5 min at 5000 rpm. The supernatant was decanted and the remaining solid was resuspended in ice cold Et_2_O and centrifugated for 5 min at 5000 rpm. The solid crude peptide salt was dissolved in MilliQ water (10 mg/mL) for purification by preparative HPLC.

### Azide-alkyne cu(I)-mediated Huisgen 1,3-dipolar cycloaddition

Fully protected peptide **10** (0.1 mmol, 1 eq) was swollen in presence of DCM under a flow of N_2_ for 10 min at RT. N_2_ was bubbled for 10 min through DMSO (2 mL) at RT, and CuBr (14.3 mg, 0.1 mmol, 1 eq.) was subsequently dissolved in the purged solvent. DCM was drained from the swollen resin and the CuBr solution was added the resin under a flow of N_2_. Sodium ascorbate (19.8 mg, 0.1 mmol, 1 eq.), 2,6-lutidine (115 µL, 1 mmol, 10 eq.) and DIPEA (173 µL, 1 mmol, 10 eq.) were dissolved in MilliQ H_2_O (2 mL) and the mixture was thereafter added to the bubbling resin. The reaction mixture was purged for 10 min under N_2_ flow before the vessel was sealed and agitated overnight at RT using a rotating mixture. The resin was washed extensively with a mixture of DMSO/isopropanol (3:5, v/v) thrice, followed by washes with DCM (3x) and DMF (3x). Upon completion of the linear peptide sequence, the peptide was treated with 20% piperidine in DMF for 30 min on a rotating-mixer at RT, of which full deprotection is monitored by Kaiser test. The resin was subsequently washed extensively with DCM (3x), MeOH (3x) and Et_2_O to yield a dried resin before cleavage from resin for 4 h on a rotating-mixer at RT using a cleavage cocktail of TFA/TIS/H_2_O (95/2.5/2.5 v/v). Ice cold Et_2_O was subsequently added to the TFA solution to yield a milky white suspension, which was subjected to centrifugation in in an Eppendorf 5804R centrifuge for 5 min at 5000 rpm. The supernatant was decanted and the remaining solid was resuspended in ice cold Et_2_O and centrifugated for 5 min at 5000 rpm. The solid crude peptide salt was dissolved in MilliQ water (10 mg/mL) for purification by preparative HPLC. Loss of the azide functional group was confirmed by Agilent carry 630 FTIR (Agilent, Santa Clara, USA).

### SETD3 expression and purification

Recombinant human SETD3 was expressed as previously described^23^. Briefly, N-terminal His6-tagged SETD3 was expressed in presence of Isopropyl β-D-1-thiogalactopyranoside (0.3 mM) overnight in *E. coli* BL21(DE3) by culturing at 13 °C, 200 rpm. SETD3 was subsequently purified by using a HisTrap FF column (5 ml) and eluted in 20 ml of elution buffer (50 mM Hepes pH 7.5, 400 mM NaCl, 10 mM KCl, 300 mM imidazole, 1 mM DTT). Overnight buffer exchange by dialysis with 500 mL dialysis buffer (20 mM Tris-HCl pH 7.5, 200 mM NaCl, 1 mM DTT and 6% sucrose) at 4 °C was followed up by two repeated sequential dialysis steps against 500 ml of dialysis buffer for 3 h at RT. Purity was evaluated with SDS-Page (> 97%). The enzyme solution was subsequently aliquoted and stored at − 70 °C. Protein concentration was determined by UV/Vis spectroscopy at using sequence specific absorbance at 280 nm with a NanoDrop 2000 spectrophotometer (Thermo Scientific, Waltham, MA, USA).

### MALDI-TOF MS enzymatic substrate scope assays

SETD3 substrate scope experiments towards βA peptides were performed by incubation of βA peptides (10 µM) in methylation buffer (25 mM Tris, 20 mM NaCl, pH = 9.0) in the presence of SAM (100 µM) and SETD3 (1 µM) in a final volume of 50 µL for 1 and 3 h at 37 ^o^C while shaking at 750 rpm in an Eppendorf Thermomixer C. The reaction was quenched by addition of 10% TFA in MilliQ H_2_O (1:1). The reactions were aliquoted and mixed with α-cyano-4-hydroxycinnamic acid dissolved in MilliQ H_2_O and ACN (1:1, v/v) with 0.1% TFA. The aliquots were loaded onto a MTP 384 polished steel target for MALDI-TOF analysis using a UltrafleXtreme and conversion to methylated product was monitored.

### MALDI-TOF MS methylation kinetics assays

Kinetics evaluation was carried out with a MALDI-TOF MS assay under steady state conditions. βA peptides (3.125–150 µM) and SAM (100 µM) were dissolved in methylation buffer (25 mM Tris, 20 mM NaCl, pH = 9.0), upon which the reaction was initiated by addition of SETD3. Reactions were incubated at 37 ^o^C and shaken at 750 rpm Eppendorf Thermomixer C for 30 min, upon which the reactions were quenched by addition of 10% TFA in MilliQ water (1:1). Reactions were aliquoted and mixed with α-cyano-4-hydroxycinnamic acid dissolved in MilliQ H_2_O and ACN (1:1, v/v) with 0.1% TFA. The aliquots were loaded onto a MTP 384 polished steel target for MALDI-TOF analysis using a UltrafleXtreme. Methylated product was quantified by determining the combined integrals of the various ionic species of the methylated product. Kinetic parameters were subsequently determined by plotting V_0_ values and actin peptide concentrations and fitting them to the Michaelis–Menten equation using GraphPad Prism software. Experiments were performed in duplicate and values are reported as mean $\:\pm\:$ standard error (SE) (Figs. S3-S4).

### MALDI-TOF MS inhibition assays

Single point inhibition screening assays were performed by dissolving βA peptides (10 or 100 µM), SAM (100 µM) and SETD3 (360 nM) in methylation buffer (25 mM Tris, 20 mM NaCl, pH = 9.0). The mixture was left for preincubation at 37 ^o^C and shaken at 750 rpm Eppendorf Thermomixer C for 20 min. Native βA (10 µM) was added and the reaction was incubated for an additional 20 min at 37 ^o^C and shaken at 750 rpm. The reactions were quenched by addition of 10% TFA in MilliQ water (1:1). Reactions were aliquoted and mixed with α-cyano-4-hydroxycinnamic acid dissolved in MilliQ H_2_O and ACN (1:1, v/v) with 0.1% TFA. The aliquots were loaded onto a MTP 384 polished steel target for MALDI-TOF analysis using a UltrafleXtreme. Residual SETD3 activity was calculated from the combined integrals of the various ionic species of the methylated native βA peptide and was subsequently normalized to a control reaction performed in absence of inhibitors. Experiments were performed in duplicate, and values are reported mean $\:\pm\:$ standard error (SE).

### Isothermal titration calorimetry

ITC experiments were performed using a MicroCal PEAQ-ITC instrument. For the titrations, βA peptides and the recombinant SETD3 enzyme were dissolved in the same ITC buffer (20 mM Tris, 200 mM NaCl, pH 8.0). The concentrations of all peptides and the protein were measured by UV on a Thermo-Fisher Nanodrop spectrophotometer in buffer using λ_max_ at 280 nm. SETD3 (30 µM) was first incubated with SAH (350 µM) for 1 h prior to the titration of βA peptides (700–850 µM). Each ITC titration consisted of 19 injections (2.0 µL) and a 0.4 µL preinjection. ITC fitting curves were processed using the One Set of Sites model in the MicroCal ITC analysis software.

### Molecular dynamics simulations

The starting conformations of SETD3 and βA peptides (PDB ID: 6ICV) were initially prepared in Maestro^31^, where protonation states and bond orders were determined. Subsequently, the structure of SETD3 and βA were exported individually. The systems’ starting coordinates and parameters were then built and combined using tleap. tleap was also used to generate starting coordinates, using the coordinates from the crystal structure as a reference. The protein/peptide systems were solvated in a TIP3P^32^ water box with a 0.150 M NaCl concentration and a 12 Å buffer distance. tleap was also used to introduce the I75K substitution, producing the βA-K75 peptide.

Simulations were performed using Amber18^33^ based on a 2 fs timestep, with GAFF^34^ parameters for SAH, and the ff14SB force field parameters for the protein and peptide. Charges for SAH and the amide linked lysine and glutamate of **9** were assigned based on electrostatic potential (ESP) fitting with Antechamber, which also assigned atom types. The ESP used in the fit was obtained with Gaussian09^35^ based on HF/6-31G* optimized structures. This ESP was then used in a RESP^36^ fitting procedure performed by Antechamber.

The simulations were performed after a short 1000 step minimization, by first heating the system to 300 K for 50 ps. The Berendsen barostat was then applied and an additional 10 ns simulation was performed to equilibrate the system, before the final 100 ns were simulated using the NPT ensemble and used for analysis. The entire simulation procedure was repeated eight times for each system. The analysis of the trajectories were performed in python using MDAnalysis^37^.

## Electronic supplementary material

Below is the link to the electronic supplementary material.

Supplementary Material 1

## Data Availability

All data generated or analysed during this study are included in this published article and its supplementary information files.
