# Supplementary material for: Examining prestructured β-actin peptides as substrates of histidine methyltransferase SETD3
Source: Sci Rep. 2024 Nov 2;14:26439. doi: 10.1038/s41598-024-76562-z (PMC11531485; doi:10.1038/s41598-024-76562-z)
Supplement: Supplementary file 1 — Supplementary Material 1 [file 41598_2024_76562_MOESM1_ESM.docx]

**Supplementary Information**

**Examining prestructured β-actin peptides as substrates of histidine methyltransferase SETD3**

Marijn N. Maas^1^, Nurgül Bilgin^1^, Laust Moesgaard^1^, Jordi C. J. Hintzen^1^, Anna Drozak^2^, Jakub Drozak^3^, Jacob Kongsted^1^ & Jasmin Mecinović^1,*^

^1^ Department of Physics, Chemistry and Pharmacy, University of Southern Denmark, Campusvej 55, 5230 Odense, Denmark.

^2^ Department of Molecular Plant Physiology, Faculty of Biology, University of Warsaw, Miecznikowa 1, 02-096 Warsaw, Poland

^3^ Department of Metabolic Regulation, Faculty of Biology, University of Warsaw, Miecznikowa 1, 02-096 Warsaw, Poland

* E-mail: mecinovic@sdu.dk

# Table of Contents

| 1 Experimental section | 3 |
| --- | --- |
| 2 Purification of βA peptides | 3 |
| 3 Characterization of βA peptides | 4 |
| 4 MALDI-TOF MS methylation supporting figures | 7 |
| 5 Isothermal titration calorimetry data | 9 |
| 6 MD simulations | 10 |

# 1 Experimental section

All reagents were obtained from commercial sources and used without further purifications. Water was purified using a Millipore Milli-Q water purification system (Merck-Millipore, Burlington, MA, USA). Fmoc-Lys(Alloc)-OH, Fmoc-Orn(Alloc)-OH, Fmoc-Dab(Alloc)-OH, Fmoc-Glu(OAll)-OH, and other Fmoc-protected amino acids were purchased from Novabiochem (Merck-Millipore). Tetrakis(triphenylphosphine)palladium(0) and phenylsilane were ordered from Sigma Aldrich. VaCo 2 lyophilizer (Zirbus Technology GmbH, Bad Grund, Germany) was used for lyophilization of pure peptides.

# 2 Purification of βA peptides

Preparative reverse-phase HPLC (RP-HPLC) was used to purify the crude peptide using a gradient elution at a constant flow rate of 10 mL/min. The parameters for the gradient using 0.1% TFA acid in MilliQ (solvent A) and 0.1% TFA acid in acetonitrile (solvent B) were as follows: Isocratic 3% B (0-3 min), gradient 3-15% B (3-12 min), gradient 15-30% B (12-17 min), gradient 30-100% B (17-19 min), isocratic 100% B (19-24 min), gradient 100-3% B (24-27 min), Isocratic 3% B (27-30 min). Analytical RP-HPLC was used to determine final peptide purity using a gradient of buffer A and buffer B from 5% B to 95% over 30 minutes at 1 mL/min on a Gemini 5μm C18 110Å LC column (Phenomenex). Analytical injections were monitored at 215 and 254 nm. Peptides with purity of 90% and higher were used in subsequent experiments. Peptide concentration was determined using sequence specific absorbance at 280 nm with a Nanodrop 2000 (Thermo Fischer Scientific, Waltham, MA, USA).

# 3 Characterization of βA-peptides

| Table S1 Characterization of βA peptides | | | | | |
| --- | --- | --- | --- | --- | --- |
| Peptide | Sequence | Formula | m/z calc. | m/z found | aHPLC rt (min) |
| 1 | TLKYPICHGCVTNWDD | C82H122N22O24S2 | 1864.1 | 1864.3 | 11.5 |
| 2 | TLKYPICHGCVTNWDD | C85H124N22O25S2 | 1918.2 | 1918.0 | 11.7 |
| 3 | TLKYPICHGCVTNWDD | C87H130N22O24S2 | 1932.3 | 1932.0 | 12.3 |
| 4 | TLKYPICHGCVTNWDD | C88H120F4N22O24S2 | 2010.2 | 2010.0 | 12.8 |
| 5 | TLKYPICHGCVTNWDD | C94H120F8N22O24S2 | 2158.2 | 2158.0 | 11.5 |
| 6 | TLKYPIEHGOrnVTNWDD | C86H129N23O26 | 1901.1 | 1901.0 | 10.9 |
| 7 | TLKYPIEHGOrnVTNWDD | C86H127N23O25 | 1883.1 | 1883.0 | 11.3 |
| 8 | TLKYPIEHGKVTNWDD | C87H131N23O26 | 1915.1 | 1915.1 | 10.9 |
| 9 | TLKYPIEHGKVTNWDD | C87H129N23O25 | 1897.1 | 1897.0 | 11.2 |
| 10 | TLKYPILys(N3)HGPraVTNWDD | C87H127N25O24 | 1907.1 | 1907.1 | 12.1 |
| 11 | TLKYPILys(N3)HGPraVTNWDD | C87H127N25O24 | 1907.1 | 1907.1 | 11.3 |
| βA | TLKYPIEHGIVTNWDD | C87H130N22O26 | 1900.1 | 1900.1 | 11.9 |


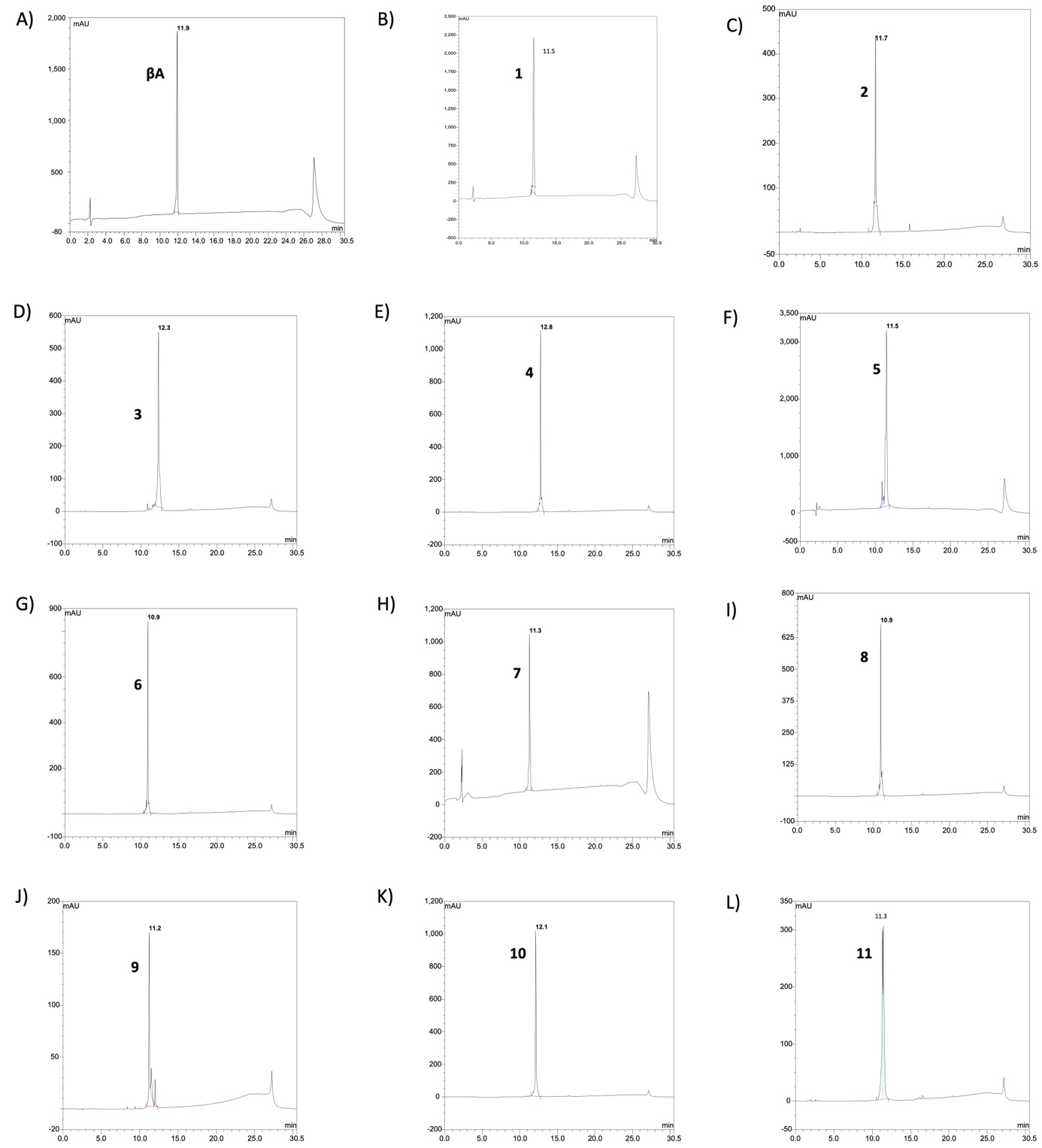


# Figure S1. A-L) Analytical HPLC of βA-peptides after RP-HPLC purification.


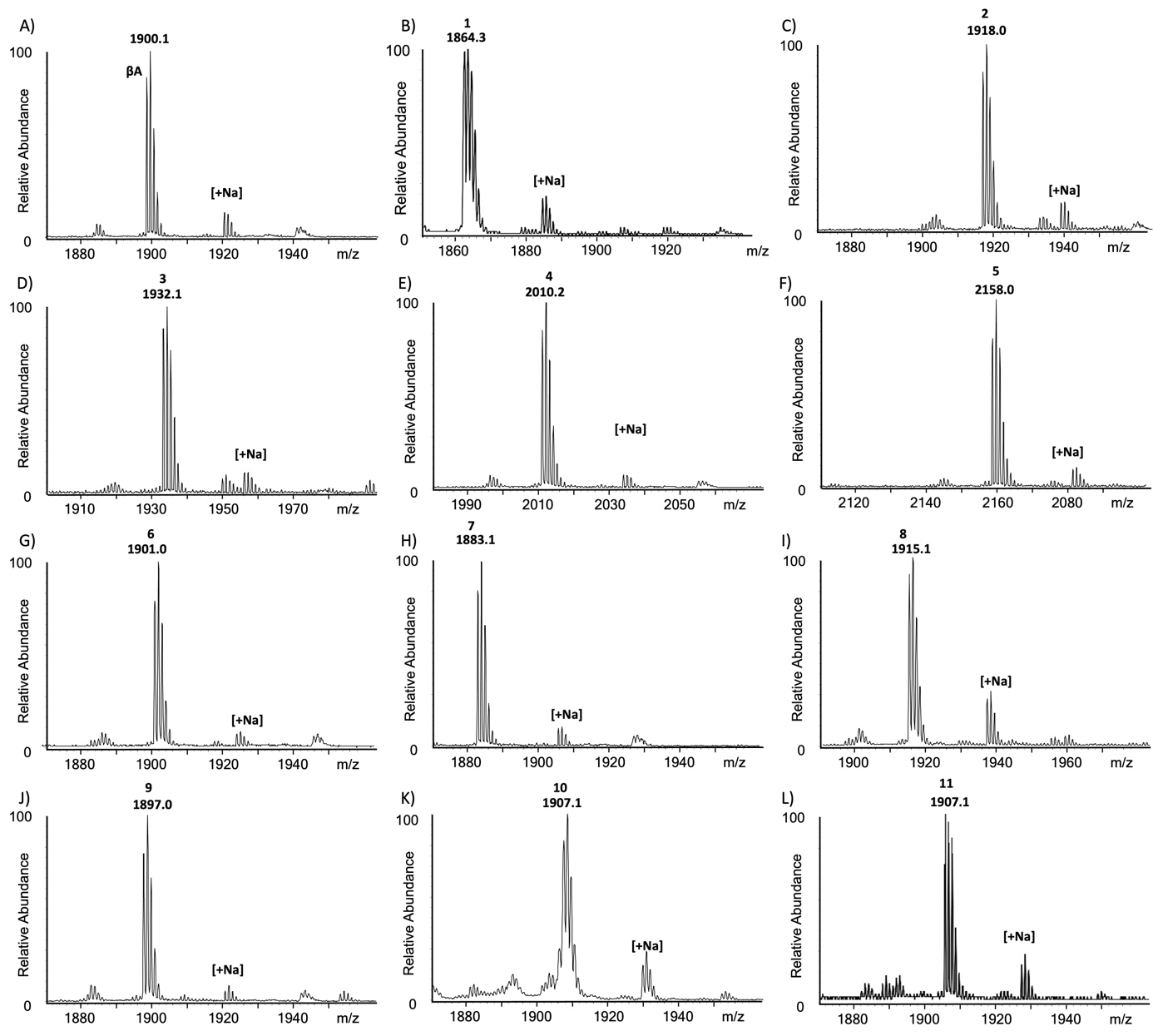


Figure S2. A-L) MALDI-TOF MS spectra of the purified βA-peptides.

# 4 MALDI-TOF MS methylation supporting figures

| **1 hour**  **** | **3 hours**  **** |
| --- | --- |
| Figure S3. Histograms depicting percentage of SETD3-catalyzed (1 $\mu$M) methylation of βA peptides (10 $\mu$M) in the presence of SAM (100 $\mu$M) in reaction buffer (25 mM Tris, 20 mM NaCl, pH = 9.0) after 1 h at 37 $℃$. N = 2. | |


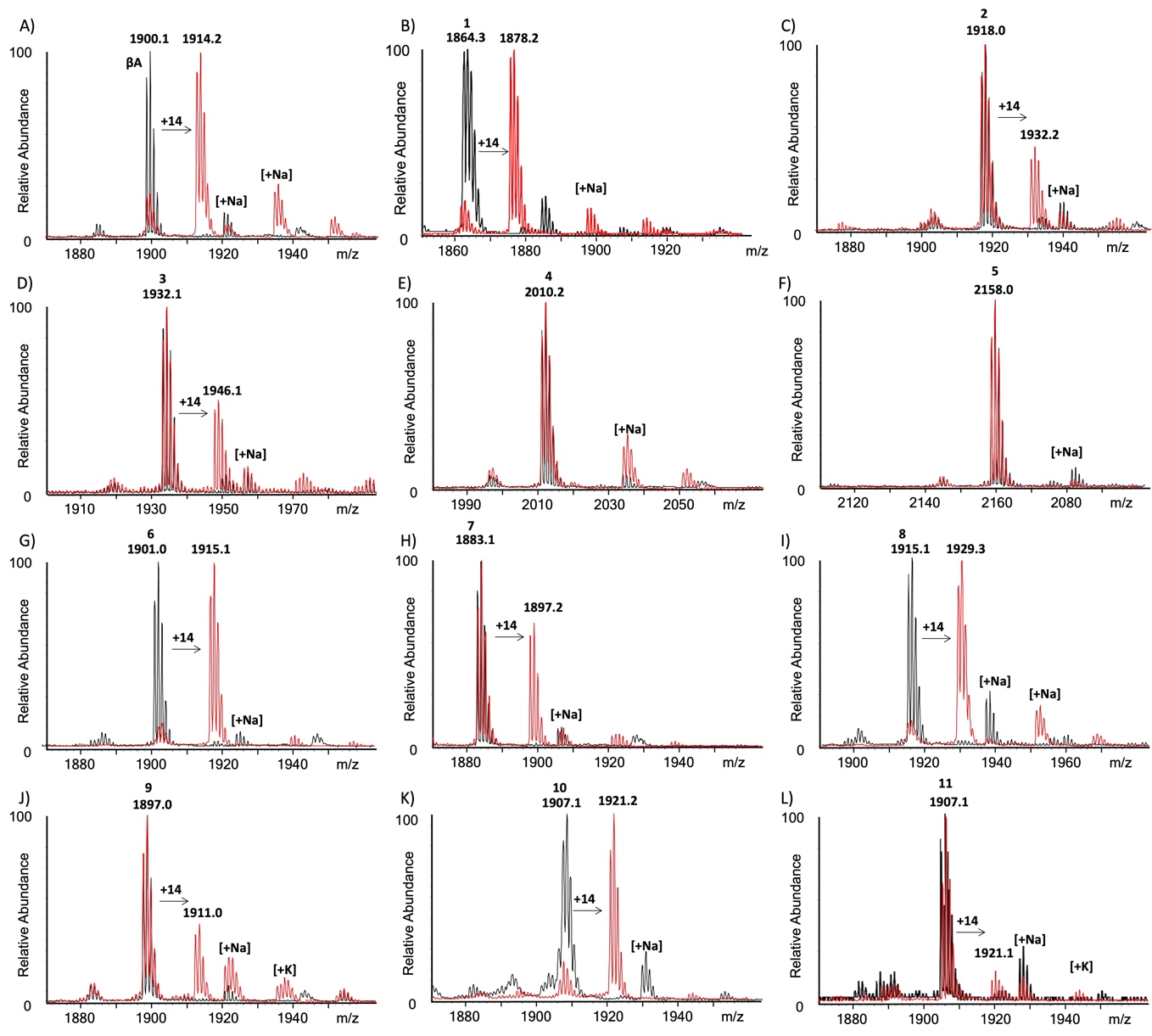


Figure S4. MALDI-TOF MS data showing SETD3-catalyzed (1 $\mu$M) methylation of βA peptides (10 $\mu$M) in the presence of SAM (100 $\mu$M) in reaction buffer (25 mM Tris, 20 mM NaCl, pH = 9.0) after 1 h at 37 $℃$. Control reactions in the absence of SETD3 are shown in black, whereas SETD3-catalyzed reactions are shown in red.

**5. Isothermal titration calorimetry data**


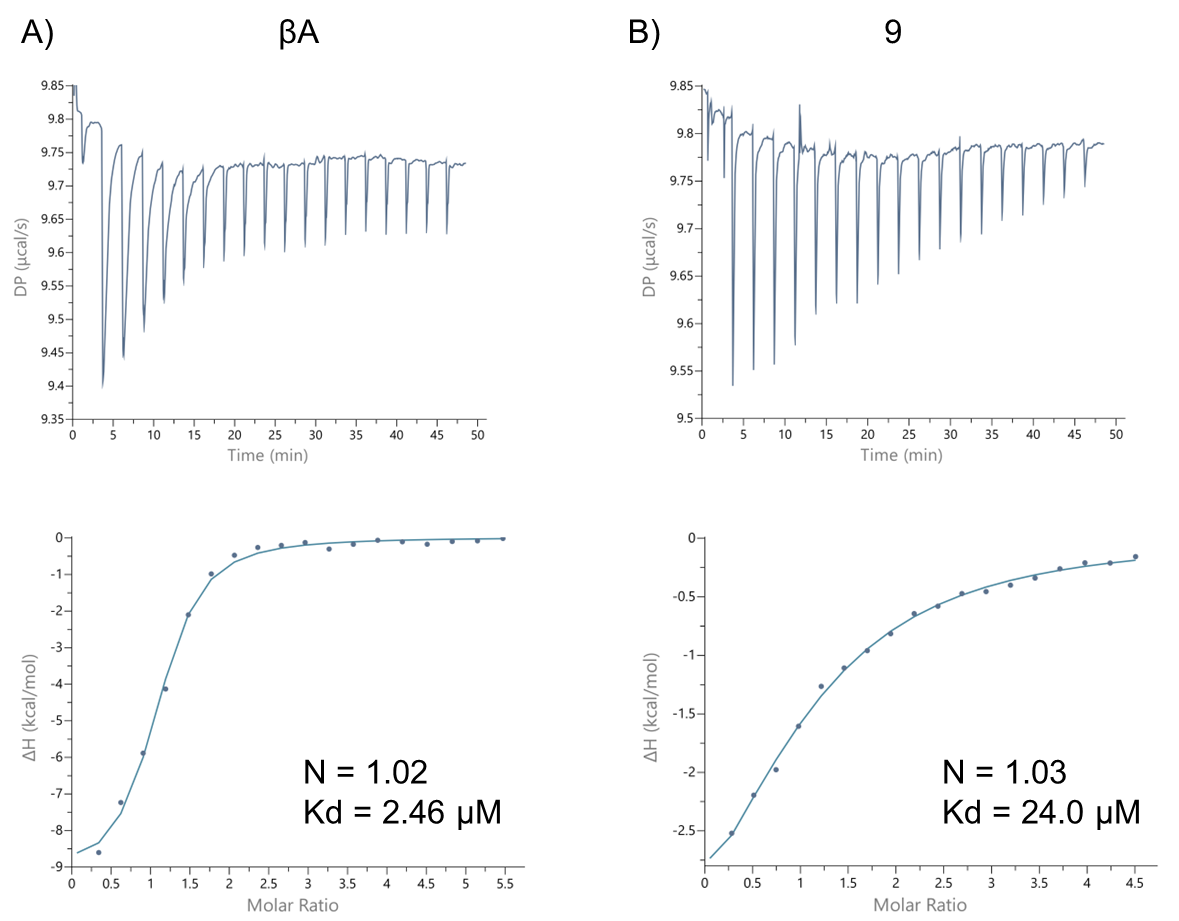


Figure S5. ITC binding curves of A) the linear βA peptide and B) the stapled peptide **9** binding to SETD3.

**6. MD simulations**


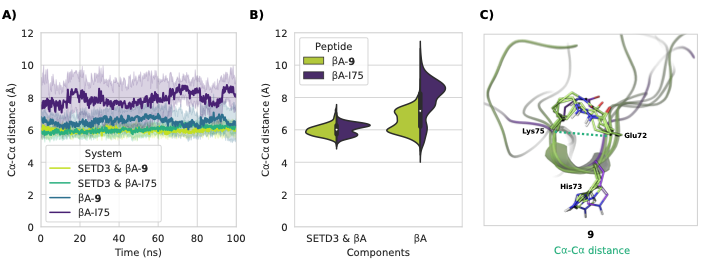


Figure S6. MD simulations of βA-I75 and βA-**9** in free and SETD3-bound forms. A) The Cα-Cα distance between Glu72 and Ile75 over time. Shaded area is the standard deviation. B) Violin-plot of the distribution of distances in A). C) Visualization of the Cα-Cα distance (the dashed line) for clustered conformations from the MD simulations of free βA-**9** (green) compared to protein bound conformation (purple).

Figure S7. The salt-bridge distances (O--N) formed by Glu72 during the MD simulations. A) The mean distance over time. Shaded area is the standard deviation. B) Violin-plot of the distribution of distances in A). C) Visualization of the salt-bridge distances of Glu72 in βA-K75 (the dashed line).

Figure S8. The Cα-Cα distance between Leu67 and Asp81 during the MD simulations. A) The mean distance over time. Shaded area is the standard deviation. B) Violin-plot of the distribution of distances in A). C) Visualization of the Cα-Cα distance (the dashed line) for clustered conformations of free βA-I75 (yellow) and βA-K75 (purple).
